# Elevated ratio of acylated to unacylated ghrelin in children and young adults with Prader–Willi syndrome

**DOI:** 10.1007/s12020-015-0614-x

**Published:** 2015-05-20

**Authors:** R. J. Kuppens, G. Diène, N. E. Bakker, C. Molinas, S. Faye, M. Nicolino, D. Bernoux, P. J. D. Delhanty, A. J. van der Lely, S. Allas, M. Julien, T. Delale, M. Tauber, A. C. S. Hokken-Koelega

**Affiliations:** Dutch Growth Research Foundation, Westzeedijk 106, 3016 AH Rotterdam, The Netherlands; Department of Pediatrics, Subdivision of Endocrinology, Erasmus University Medical Center-Sophia Children’s Hospital, Rotterdam, The Netherlands; Unité D’endocrinologie, Obésité, Maladies Osseuses, Génétique et Gynécologie Médicale, Centre de Référence du Syndrome de Prader-Willi, Hôpital des enfants, Toulouse, France; Axe pédiatrique du CIC 9302/INSERM. Hôpital des enfants, Toulouse, France; Division of Pediatric Endocrinology, Hôpital Femme-Mère-Enfant, University of Lyon, Bron/Lyon, France; Department of Internal Medicine, Erasmus University Medical Center, Rotterdam, The Netherlands; Alizé Pharma, 69130 Ecully, France; INSERM U1043, Centre de Physiopathologie de Toulouse Purpan, Université Paul Sabatier, Toulouse, France

**Keywords:** Prader–Willi syndrome, Ghrelin, Acylated ghrelin, Unacylated ghrelin, AG, UAG, AG/UAG ratio

## Abstract

Prader–Willi syndrome (PWS) is characterized by a switch from failure to thrive to excessive weight gain and hyperphagia in early childhood. Hyperghrelinemia may be involved in the underlying mechanisms of the switch. The purpose of this study is to evaluate acylated ghrelin (AG) and unacylated ghrelin (UAG) levels in PWS and investigate their associations with hyperphagia. This is a cross-sectional clinical study conducted in three PWS expert centers in the Netherlands and France. Levels of AG and UAG and the AG/UAG ratio were determined in 138 patients with PWS (0.2–29.4 years) and compared with 50 age-matched obese subjects (4.3–16.9 years) and 39 healthy controls (0.8–28.6 years). AEBSF was used to inhibit deacylation of AG. As a group, PWS patients had higher AG but similar UAG levels as healthy controls (AG 129.1 vs 82.4 pg/ml, *p* = 0.016; UAG 135.3 vs 157.3 pg/ml, resp.), resulting in a significantly higher AG/UAG ratio (1.00 vs 0.61, *p* = 0.001, resp.). Obese subjects had significantly lower AG and UAG levels than PWS and controls (40.3 and 35.3 pg/ml, resp.), but also a high AG/UAG ratio (1.16). The reason for the higher AG/UAG ratio in PWS and obese was, however, completely different, as PWS had a high AG and obese a very low UAG. PWS patients without weight gain or hyperphagia had a similar AG/UAG ratio as age-matched controls, in contrast to those with weight gain and/or hyperphagia who had an elevated AG/UAG ratio. The switch to excessive weight gain in PWS seems to coincide with an increase in the AG/UAG ratio, even prior to the start of hyperphagia.

**Electronic supplementary material:**

The online version of this article (doi:10.1007/s12020-015-0614-x) contains supplementary material, which is available to authorized users.

## Introduction

Prader–Willi syndrome (PWS) is a neurogenetic disorder caused by the lack of expression of the paternally derived genes on chromosome 15 at locus q11–q13 [1]. Clinical findings change when children become older: infancy is characterized by poor feeding, failure to thrive, and muscular hypotonia, while hyperphagia with impaired satiety, obesity, short stature, psychomotor delay, and behavioral problems are prominent during childhood and adulthood [2, 3]. The mechanism behind the switch from failure to thrive to excessive weight gain and hyperphagia in early childhood is not yet known, but hyperghrelinemia might be involved [4, 5].

Most children with PWS nowadays receive growth hormone (GH) treatment, which counteracts the clinical course of obesity and improves the metabolic profile, leading to better lipid levels, higher adiponectin levels, and lower systolic blood pressure [6, 7]. However, GH treatment does not solve the problem of hyperphagia.

The appetite-stimulating hormone ghrelin has an acylated and an unacylated form in circulation [8–10]. Acylated ghrelin (AG) is known to be diabetogenic and has many actions such as stimulating appetite and inducing a positive energy balance, which can lead to weight gain [11–14]. Intravenous AG administration in healthy volunteers increased food intake and appetite [15], suggesting that the hyperphagia in PWS might be associated with increased ghrelin levels. For a long time, unacylated ghrelin (UAG) was considered to be an inactive degradation product of AG. Currently, there is increasing evidence that UAG has also distinct actions [16]. It is reported that UAG has protective effects on beta cells, endothelial progenitor cells, and muscle cells, and UAG seems to improve glycemic control [14]. In addition, UAG acts as a functional inhibitor of AG and it was reported to suppress ghrelin levels in humans [16, 17]. This suggests a crucial role for the ratio of AG and UAG levels (AG/UAG ratio) in maintaining weight balance.

Ghrelin can be determined in blood samples, but AG is unstable and is rapidly deacylated to UAG through the action of esterases [18, 19]. Thus, reliable ghrelin determination needs the immediate addition of an esterase inhibitor at the time of blood collection. In patients with PWS, hyperghrelinemia has been reported, but in these studies, samples were not treated in this way [4, 5, 20–23].

We formulated three hypotheses. Children and young adults with PWS have (1) higher AG levels and lower UAG levels than healthy and obese controls; (2) increasing AG and decreasing UAG levels with the rise of the nutritional phases, resulting in increased AG/UAG ratios in the higher nutritional phases; and (3) a higher AG/UAG ratio in the presence of weight gain and/or hyperphagia and a normal AG/UAG ratio in the absence of weight gain and/or hyperphagia, compared with age-matched healthy controls.

Therefore, a cross-sectional study was conducted in which we measured the plasma levels of AG and UAG in children and young adults with PWS and compared these levels with those of obese and healthy controls. AEBSF, an inhibitor of deacylation of AG, was immediately added to all blood samples. In patients with PWS, we investigated the associations between AG and UAG ghrelin levels and the following factors: age, BMI, genotype, eating behavior and food intake. In addition, we investigated whether the switch from failure to thrive to excessive weight gain and hyperphagia is associated with a change in the AG/UAG ratio.

## Subjects and methods

### Subjects

The study group consisted of 138 children and young adults with PWS, either participating in the Dutch PWS studies coordinated by the Dutch Growth Research Foundation, or followed at the PWS reference center in Toulouse or Children’s Hospital in Lyon. PWS was genetically confirmed in all patients. One hundred and seven patients (77.5 %) were treated with GH, and the others had not yet started with GH or had reached final height without possibilities to continue GH treatment or the parents refused the GH treatment. Three patients with PWS had diabetes mellitus type 2 (DM2) and all were treated with metformin. As their ghrelin levels were similar as in the total PWS group, we did not exclude them from analyses. None of the healthy or obese controls had DM2.

Plasma ghrelin levels of subjects with PWS (PWS) were compared with 50 obese subjects (obese) and 39 healthy controls (controls). Obese and controls suffering from any systemic illness, growth disorder, syndrome, or having dysmorphic features were excluded. Obese children had a BMI >+2 SDS and were regularly seen in the outpatient department of the pediatric endocrinology unit in Toulouse. Healthy controls were children and young adults with a normal BMI, who underwent a minor surgical procedure at Erasmus Medical Center in Rotterdam. Normal BMI was defined as a BMI between −2 SDS and +2 SDS [24].

Standing height was measured with a calibrated Harpenden stadiometer or, when appropriate, supine length with a Harpenden infantometer (Holtain Ltd). Weight was determined on a calibrated scale (Servo Balance KA-20-150S; Servo Berkel Prior) and BMI was calculated. Height, weight and BMI were expressed as SDS, adjusted for age and sex. The Dutch reference data were used for the Dutch children and young adults [24, 25], and the French reference data for height and weight and the Cole BMI reference data were used for the French patients [26, 27]. All SDS values were calculated with Growth Analyser (version 4.0; www.growthanalyser.org).

The Medical Ethics Committees of the 3 participating centers approved the study. Written informed consent was obtained from parents of PWS. For obese and controls, written informed consent was obtained from themselves and, if they were younger than 18 years, also from their parents or custodians.

### Eating behavior

The nutritional phases according to Miller were used to score the eating behavior of the subjects with PWS [3]: 1a Hypotonia with difficulty feeding, 1b No difficulty feeding and growing appropriately on growth curve, 2a Weight increasing without an increase in appetite or excessive calories, 2b Weight increasing with an increase in appetite or excessive calories, 3 Hyperphagia, feels rarely full, and 4 Appetite no longer insatiable. For each subject with PWS, the nutritional phase was assessed by the multidisciplinary teams or independently by two observers who knew them very well (physician and nurse) [3]. In case of disagreement, the case was discussed until consensus was reached. Subjects with PWS without weight gain or hyperphagia, defined as being in nutritional phase 1a or 1b, and subjects with weight gain and/or hyperphagia, defined as being in phases 2a, 2b, and 3, were compared with age-matched controls.

### Collection of blood and plasma preparation

In children >2 years, blood samples were collected in the morning after a 12-h overnight fast. Infants <2 years were fasted for at least 5-h. To stabilize the plasma ghrelin levels, blood samples were collected in EDTA tubes, and 4-(2-aminoethyl)benzenesulfonyl fluoride hydrochloride (AEBSF, Sigma-Aldrich Chemicals) was added to a concentration of 2 mg/ml at the time of collection. Blood was centrifuged at 4 °C to prepare plasma, which was quickly frozen on dry ice. Samples were stored at −80 °C and assayed within 3 months following collection.

### Assays

Plasma AG and UAG levels were assessed in duplicate (10-50 µL per well) in one laboratory using two-step double-antibody sandwich EIAs, obtained from SPIBio (Bertin Pharma, France; A05306 and A05319, resp.). Assays were performed according to manufacturer’s instructions. In summary, standards, quality controls, and samples were incubated in the plate for 2 h at room temperature without tracer. After a 3× wash, tracer antibody was added and incubated for 2 h at room temperature. Following a 5× wash, Ellman’s reagent was added and incubated for approximately 45 min until satisfactory color development. Finally, the absorbance was measured at 405 nm using a VictorX4 plate reader (PerkinElmer, Groningen, Netherlands).

Data were analyzed using Graphpad Prism 5 (La Jolla, California). A sigmoidal third-order (cubic) polynomial fitting was used to determine concentrations from the calibration curves. This resulted in *r*^2^ values >0.99 in the majority of the assays. Intra-assay coefficient of variations (CVs) for AG and UAG were 8.2 and 11.4 %, and interassay CVs for AG and UAG were 3.9 and 11.0 %. CVs were determined over 10 and 9 assays for AG and UAG, resp. Samples had inter-duplicate CV of <20 % for both AG and UAG. The AG/UAG ratio was computed as AG divided by UAG.

In Rotterdam, insulin levels were assessed using the Immulite 2000 assay (Siemens Healthcare Diagnostics). Interassay CV was 4.4 %. Serum glucose levels were determined using the Hitachi 917 (Hitachi Device Development Center), detecting glucose levels between 0 and 42 mmol/l. Serum IGF-I levels were assessed using the IDS-iSYS (Immunodiagnostic Systems). The intra-assay CV was <6.0 % and the interassay CV was <2.1 %. In France, insulin and glucose were enzymatically assessed on the Beckman AU 2700 (Beckman Coulter Inc) and serum IGF-I levels were measured using IRMA assay from Immunotech. The intra-assay CV was <6.3 % and the interassay CV was <8.8 %. For Rotterdam and France, homeostasis model assessment of insulin resistance (HOMA-IR) was performed using the model HOMA-IR = [fasting insulin (mU/l) × fasting glucose (mmol/l)]/22.5 [28].

### Statistics

Statistical analysis was performed by the Statistical Package for Social Sciences (version 20.0; SPSS, Chicago, IL). Data are expressed as median [interquartile range (IQR)]. Differences between the groups were calculated using Kruskal–Wallis tests when comparing three groups and Mann–Whitney *U* tests when comparing two groups. Patients with PWS in each nutritional phase were compared with age-matched healthy controls. Obese PWS, defined as BMI >+2 SDS, were compared with obese controls. AG and UAG levels and AG/UAG ratios were log-transformed (natural logarithm), as they were not normally distributed. In PWS patients, we cross-sectionally assessed linear correlations between ghrelin levels and other parameters using Spearman’s rho correlation coefficient (*ρ*). As levels of AG and UAG decreased with age, linear regression analysis was used to compare groups with adjustment for age. The AG/UAG ratio was not adjusted for age, as it remained stable across ages. Linear regression analysis was used to analyze correlations with adjustment for parameters such as age and gender. Regression coefficients are presented as percentages for better interpretation of the results. A positive value indicates that the dependent variable is increased by that % for every unit increase of the independent variable. Differences were considered significant if the *p* value was <0.05.

## Results

### Clinical characteristics

Median [IQR] age of PWS was 9.9 [4.1–14.9] years (Table 1). There was no significant difference in age between the 138 PWS (61 boys), 39 controls (13 boys), and 50 obese (16 boys). Height, weight and BMI were significantly different between the three groups and highest in obese (*p* < 0.001). Fasting insulin and HOMA-IR were higher in obese (*p* < 0.001 and *p* = 0.001, resp.), while fasting glucose and IGF-I SDS were highest in PWS (*p* = 0.043 and *p* < 0.001, resp.).

**Table 1:** Baseline characteristics of 138 PWS, 39 healthy controls and 50 obese subjects

	PWS *n* = 138	Controls *n* = 39	Obese *n* = 50	
	Median (IQR)	Median (IQR)	Median (IQR)	p*
Age (years)	9.9 (4.1 to 14.9)	7.3 (3.6 to 13.6)	9.8 (7.9 to 13.0)	0.350
Weight for age (SDS)	0.8 (−0.6 to 2.4)	−0.2 (−0.8 to 0.5)	5.9 (4.5 to 7.0)	**<0.001**
Height for age (SDS)	−0.3 (−1.3 to 0.6)	−0.1 (−0.8 to 0.4)	1.3 (0.8 to 2.5)	**<0.001**
BMI for age (SDS)	1.0 (−0.2 to 2.1)	−0.2 (−0.8 to 0.8)	2.8 (2.6 to 3.1)	**<0.001**
Fasting glycemia (mmol/l)	4.5 (4.0 to 5.0)		4.3 (3.9 to 4.7)	**0.043**
Fasting insulin (pmol/l)	62.5 (34.7 to 90.3)		100.7 (76.4 to 123.3)	**<0.001**
HOMA-IR	1.8 (1.0 to 2.8)		2.6 (1.9 to 3.6)	**0.001**
IGF-I (SDS)	0.7 (0.7 to 1.7)		−0.5 (−1.2 to 0.3)	**<0.001**

Bold values are statistically significant (*p* < 0.05)

* *p* value between the three groups; 138 PWS, 39 healthy controls and 50 obese controls

PWS was genetically confirmed by an abnormal methylation test in all subjects with PWS. In 131 (94.9 %) subjects, the genetic subtype was known; seventy (50.7 %) had a deletion, 55 (39.9 %) a uniparental maternal disomy (mUPD), and 6 (4.3 %) an imprinting center mutation. One hundred and seven PWS used GH (77.5 %) with a median dose of 0.85 mg/m^2^/day [0.61–1.0] (≈0.028 mg/kg/day). Median age at start of GH treatment was 1.9 [1.1–4.3] years and median duration of GH treatment was 7.0 [3.2–9.1] years.

### Ghrelin levels in the three groups

**Fig. 1:**
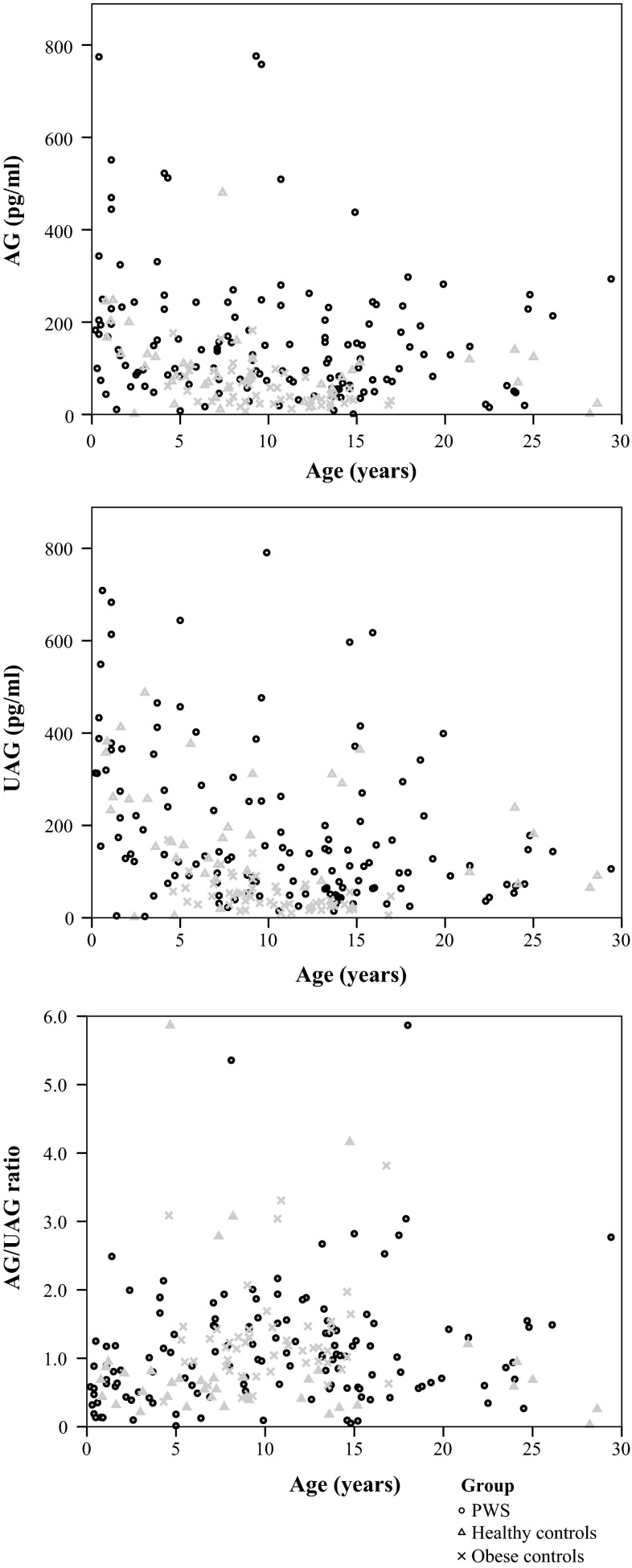
Ghrelin levels versus age for the three groups. These figures show the of AG and UAG levels and the AG/UAG ratio of children and young adults with PWS in* black dots*, of healthy controls in* gray triangles* and of obese controls in* gray crosses*

Median [IQR] AG was significantly higher in the PWS group (129.1 [67.1–227.9] pg/ml) than in controls (82.4 [56.3–130.4] pg/ml, *p* = 0.016). UAG was similar in PWS and controls. As a result, the AG/UAG ratio was significantly higher in PWS than in controls (*p* = 0.001) (Table 2; Fig. 1).

**Table 2:** Ghrelin levels of the three groups

	PWS	Controls	Obese	*p* between groups*	PWS versus controls*	PWS versus obese*	Obese versus controls*
	*n* = 138	*n* = 39	*n* = 50				
Parameter	Median (IQR)	Median (IQR)	Median (IQR)				
AG (pg/ml)	129.1 (67.1–227.9)	82.4 (56.3–130.4)	40.3 (26.4–82.5)	**<0.001**	**0.016**	**<0.001**	**0.001**
UAG (pg/ml)	135.3 (66.0–284.2)	157.3 (79.3–261.0)	35.8 (26.0–64.4)	**<0.001**	0.868	**<0.001**	**<0.001**
AG/UAG ratio	1.00 (0.57–1.49)	0.61 (0.37–0.81)	1.16 (0.92–1.43)	**<0.001**	**0.001**	0.069	**<0.001**

Bold values are statistically significant (*p* < 0.05)

* *p* value between the groups

In PWS, both AG and UAG levels were significantly higher than in obese (both *p* < 0.001), as obese had the lowest AG and UAG levels of the three groups. Both PWS and obese had a significantly higher AG/UAG ratio than controls. The reason for the higher AG/UAG ratios in PWS and obese was, however, different. PWS had high AG levels, resulting in this higher AG/UAG ratio, while obese had low AG levels with even lower UAG levels.

In all three groups, both AG and UAG levels decreased with age, but the AG/UAG ratio remained stable.

### Ghrelin in nutritional phases in PWS

Of the 138 PWS who were classified according to the nutritional phases of Miller [3], 13 patients were in phase 1a, 37 in phase 1b, 12 in phase 2a, 44 in phase 2b, 31 in phase 3, and 1 in phase 4 (Table 3). The median age of the children with PWS in nutritional phase 1b was 8.9 years, while the median age of the children in nutritional phase 2a was 3.5 years younger, namely 5.3 years. It shows that the rise of the nutritional phases was not in line with an older age. A considerable number of older subjects with PWS were still in nutritional phase 1b. Parallel with the rise of the nutritional phases, children with PWS in the higher nutritional phases had a higher BMI, ranging from −2.0 SDS in phase 1a to +3.2 SDS in phase 4.

**Table 3:** Age, BMI, and ghrelin levels per nutritional phase

		Age (years)		BMI (SDS)		AG (pg/ml)		UAG (pg/ml)		AG/UAG ratio	
	*n*	Median	Range	Median	p*	Median	p*	Median	p*	Median	p*
PWS	138	9.9	0.2–29.4	1.0	**<0.001**	129.1	**0.016**	135.3	0.868	1.00	**0.001**
Nutritional phase 1a	13	0.5	0.2–4.1	−2.0	**0.001**	182.6	1.000	350.8	0.521	0.57	0.837
Nutritional phase 1b	37	8.9	0.6–22.3	−0.6	0.167	161.0	**0.022**	252.0	0.180	0.72	0.225
Nutritional phase 2a	12	5.3	1.5–9.3	0.9	**<0.001**	117.2	0.977	125.5	0.413	1.19	0.243
Nutritional phase 2b	44	11.2	2.2–29.4	1.6	**<0.001**	114.4	**0.020**	120.1	0.848	1.05	**0.002**
Nutritional phase 3	31	14.5	4.7–26.1	2.7	**<0.001**	99.2	0.217	73.1	0.165	1.26	**0.005**
Nutritional phase 4	1	14.6		3.2		56.1		596.8		0.09	
Healthy controls	39	7.3	0.8–28.6	−0.2		82.4		157.3		0.61	
Obese controls	50	9.8	4.3–16.9	2.8	**<0.001**	40.3	**0.001**	35.8	**<0.001**	1.16	**<0.001**

Bold values are statistically significant (*p* < 0.05)

* Compared with age-matched healthy controls (HC). Phase: 1a PWS *n* = 13 versus HC *n* = 8 (median age 1.4 years, median BMI -0.1 SDS), 1b PWS *n* = 37 versus HC *n* = 26 (8.5 years, −0.1 SDS), 2a PWS *n* = 12 versus HC *n* = 12 (5.2 years, −0.9 SDS), 2b PWS *n* = 44 versus HC *n* = 29 (9.0 years, 0.0 SDS), 3 PWS *n* = 31 verus HC *n* = 18 (13.9 years, 0.0 SDS)

### Ghrelin levels and the switch in eating behavior in PWS

Both AG and UAG levels of PWS patients decreased with the rise of the nutritional phases (Table 3, supplemental figure). Between phase 1b and phase 2a, AG levels decreased from 161.0 to 117.2 pg/ml and UAG levels decreased from 252.0 to 125.5 pg/ml, but probably due to the low number in phase 2a, this did not reach statistical significance. Subsequently, the AG and UAG levels of the patients in nutritional phase 2a, 2b, and 3 remained consistently low. The AG/UAG ratio showed a marked increase between phase 1b and phase 2a and then remained consistently high in phases 2a, 2b, and 3. This shows that the elevated AG/UAG ratio is already present in phase 2a, prior to phases 2b and 3 in which the hyperphagia occurs.

**Fig. 2:**
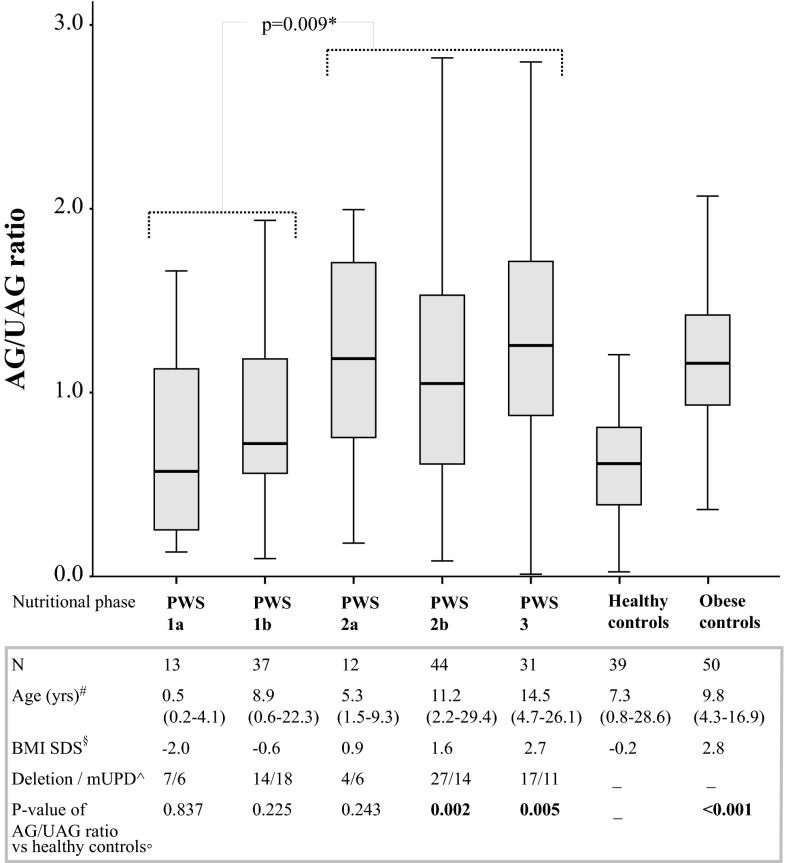
AG/UAG ratio of PWS per nutritional phase and of healthy and obese controls. This* boxplot* shows the AG/UAG ratio of children and young adults with PWS in the five nutritional phases and of healthy controls and obese controls. The lower boundary is the 25th percentile and the upper boundary the 75th percentile. The line in the* box* represents the median. Lines are drawn from the smallest to the largest observed value that is not an outlier. #Median (range) age in years. ^§^Median BMI SDS. ^Number of PWS patients with deletion and mUPD. °*P* value of AG/UAG ratio of the subjects in each nutritional phase compared with age-matched healthy controls. *All subjects in phase 1a and 1b combined and compared with all subjects in phase 2a, 2b, and 3 combined

Differences in ghrelin levels between nutritional phase 1b and 2a did not reach statistical significance. As ghrelin levels were similar in phase 2a, 2b, and 3, these nutritional phases were combined. Eighty-seven PWS had weight gain and/or hyperphagia (nutritional phase 2a, 2b, and 3). These 87 PWS had significantly higher AG/UAG ratios than the 50 PWS who did not have weight gain or hyperphagia (nutritional phases 1a and 1b) (*p* = 0.009) (Fig. 2). This shows that the switch to weight gain and/or hyperphagia seems to occur simultaneously with the increase in the AG/UAG ratio.

### Ghrelin levels in PWS and age-matched controls

While the AG/UAG ratio was similar in PWS in phase 1a and 1b as in age-matched controls, the AG/UAG ratio was significantly higher in nutritional phase 2b and 3 than in age-matched controls. The AG/UAG ratio in PWS in phase 2a was similar as in phase 2b and 3, but was not significantly higher than age-matched controls, probably due to the low number in phase 2a (Table 3; Fig. 2).

### Non-obese PWS vs healthy controls and obese PWS vs obese controls

In an additional analysis, we compared 100 non-obese PWS with a median [IQR] BMI of +0.4 [−0.9 to 1.2] SDS with 39 healthy controls (BMI −0.2 SDS [−0.8 to 0.8]). The results were comparable with the data of the total group (Table 4). The AG levels were significantly higher in non-obese PWS than in healthy controls (median 140.1 vs 82.4 pg/ml, *p* = 0.005), while the UAG levels were similar (median 150.7 vs 157.3 pg/ml, NS), resulting in a significantly higher AG/UAG ratio in non-obese PWS (0.89 vs 0.61, *p* = 0.003).

**Table 4:** Comparison between non-obese PWS and healthy controls, and obese PWS and obese controls

		Gender	Age (years)	BMI (SDS)	AG (pg/ml)	UAG (pg/ml)	AG/UAG ratio
	n	m/f	median	median	median	median	median
Non-obese PWS (BMI < 2SDS)	100	43/57	8.9	0.4	140.1	150.7	0.89
Healthy controls	39	13/26	7.3	−0.2	82.4	157.3	0.61
Non-obese PWS vs healthy controls	139	NS	NS	NS	**0.005**	NS	**0.003**
Obese PWS (BMI > 2SDS)	38	18/20	13.5	2.7	100.1	94.9	1.08
Obese controls	50	16/34	9.8	2.8	40.3	35.8	1.16
Obese PWS vs Obese controls	88	NS	**0.013**	NS	**<0.001**	**<0.001**	NS

Bold values are statistically significant (*p* < 0.05)

We also compared the ghrelin levels of 38 obese PWS with a median [IQR] BMI of +2.7 SDS [2.4–3.2] with 50 obese controls (BMI +2.8 SDS [2.6–3.1]). Both AG and UAG levels were significantly higher in obese PWS than in obese controls (median 100.1 and 94.9 pg/ml vs 40.3 and 35.8 pg/ml resp., both *p* < 0.001, even after adjustment for age), but the AG/UAG ratio was similar in both groups (1.08 vs 1.16, *p* = 0.730).

### Associations between ghrelin levels and clinical characteristics and HOMA-IR in PWS

There were no significant differences in AG and UAG levels and AG/UAG ratio between boys and girls, or between patients with a deletion and an mUPD. AG was inversely associated with age (*p* = 0.012), weight SDS (*p* = 0.004) and BMI SDS (*p* = 0.001), and similar inverse associations were found between UAG and age, weight SDS and BMI SDS (all *p* < 0.001). Since both AG and UAG decreased with age, the association analyses were adjusted for the variables age and gender. Higher BMI SDS was associated with lower AG and UAG levels, also after correction for age and gender (not shown). If the BMI SDS would increase with 1 SDS, the AG level would decrease by 22.8 % and the UAG by 21.0 %. The association between BMI SDS and AG/UAG ratio was not significant.

AG and UAG were both inversely associated with fasting insulin (*p* = 0.023 and *p* < 0.001, resp.), and UAG with HOMA-IR (*p* = 0.004). After adjustment for the variables age and gender, these associations remained significant (*p* = 0.048 and *p* = 0.031 for insulin, *p* = 0.050 for HOMA-IR). The AG/UAG ratio was not associated with insulin or HOMA-IR.

### Associations between ghrelin and IGF-I levels in PWS

IGF-I levels were measured in 124 PWS of which 103 (83.1 %) were treated with GH. IGF-I levels, adjusted for age and gender, were higher in the GH-treated versus untreated patients with PWS (*p* < 0.001). The AG and UAG levels and AG/UAG ratio were not different between the GH-treated and untreated patients with PWS (*p* = 0.423 for AG, *p* = 0.374 for UAG and *p* = 0.337 for AG/UAG ratio).

After adjustment for age and gender, IGF-I was inversely associated with AG and UAG (*p* = 0.011 and *p* = 0.008), but there was no significant association between IGF-I and the AG/UAG ratio (*p* = 0.993).

### Obese and controls

In healthy and obese controls, no significant differences in ghrelin levels between boys and girls were found. Younger age was associated with higher levels of AG and UAG (*p* < 0.001 and *p* = 0.001, resp), but the AG/UAG ratio remained stable across age. BMI SDS was inversely correlated with AG and UAG levels (both *p* < 0.001), but positively with the AG/UAG ratio (*p* < 0.001).

## Discussion

Our study shows that AG levels are significantly higher in PWS patients than in controls. In contrast to our expectations, UAG levels in PWS were similar to those in controls. This resulted in a significantly higher AG/UAG ratio in PWS than in controls. Remarkably, our study shows that PWS patients in nutritional phase 2a, 2b, and 3, thus with weight gain and/or hyperphagia, had a higher AG/UAG ratio than those in nutritional phase 1a or 1b, without weight gain or hyperphagia, whose AG/UAG ratios were similar to age-matched controls.

In our large study group, we measured levels of acylated and unacylated ghrelin separately using double-antibody sandwich ELISAs specific for each isoform. This approach prevents detection of inactive peptide fragments in the samples. We also inhibited the deacylation of AG to UAG by adding AEBSF to the blood samples [18, 19]. Many previous studies used radioimmunoassays for total ghrelin which detect both full-length, as well as inactive fragments, of both ghrelin isoforms [4, 5, 20, 29–31], or samples were not stabilized with an esterase inhibitor to prevent deacylation of AG [32, 33]. As a result, our data cannot be compared with those in earlier studies.

Both PWS and obese had a significantly higher AG/UAG ratio than healthy controls. The reason for the higher AG/UAG ratio in both groups was, however, completely different. While PWS had higher AG levels with normal UAG levels, obese controls had low AG levels with even lower UAG levels. Based on the higher BMI in PWS patients, one would have expected lower AG and UAG levels like in obese subjects. This might indicate that the abnormalities of the ghrelin system are specific for PWS. Only two other studies reported AG or UAG levels in PWS and compared them with obese controls, but neither study added an inhibitor of AG degradation to the blood samples. Both demonstrated significantly higher AG levels in PWS than in obese controls [32, 33]. The ratio between AG and UAG was not presented. Paik et al. did not report a significant difference in UAG levels between PWS and obese controls [33]. A possible explanation for these different observations might be the method that was used to collect the blood samples.

In contrast to our expectation, the median age of patients with PWS in nutritional phase 1b was 3.5 years higher than in phase 2a. Although hyperphagia is a constitutive marker of PWS, we found in our large group that several older patients were still in nutritional phase 1b, thus without weight gain or hyperphagia. An explanation might be that the patients in our group had an earlier diagnosis with earlier attention for diet, physical exercise and GH treatment starting at a young age [34], although it is not proven that this approach can prevent hyperphagia. Parallel to the rise of the nutritional phases, we found an increasing BMI, supporting that the nutritional phases were correctly attributed.

Our results confirm our hypothesis that patients with PWS without hyperphagia have a similar AG/UAG ratio as age-matched controls, while patients with PWS with weight gain and/or hyperphagia have an AG/UAG ratio higher than that of age-matched controls. In nutritional phase 2a, children with PWS gain weight without a change in appetite or caloric intake. Phase 2b is associated with weight gain and an increased interest in food, and phase 3 is characterized by hyperphagia, typically accompanied by food-seeking and lack of satiety [3]. So in nutritional phases 2a, 2b, and 3, the switch to the typical weight and eating problems of PWS has already occurred. We found that the AG/UAG ratio of children with PWS is already increased in nutritional phase 2a, when there is only weight gain but no hyperphagia. There is a considerable change in the AG/UAG ratio between phase 1b and phase 2a and the AG/UAG ratio remained at a similar high level in phases 2b and 3. Compared to age-matched controls, the AG/UAG ratio in phase 2a, 2b, and 3 was higher, although not significantly in phase 2a, probably due to the low number of patients in phase 2a. This considerable change in AG/UAG ratio between phase 1b and higher nutritional phases is in line with the switch to weight gain followed by hyperphagia, which happens in the same period and supports the hypothesis that ghrelin might be involved in this. Whether this modification is the cause or the consequence of the switch in the nutritional phases cannot be unraveled by our study. Delhanty et al. suggested that UAG is a functional inhibitor of AG which might suppress AG levels in humans [16, 17]. Elaborating on this idea, the ratio between AG and UAG levels (AG/UAG ratio) might be a more important parameter than individual AG and UAG levels. If this hypothesis is correct, it could be that in patients with PWS without weight gain and/or hyperphagia, in which the AG/UAG ratio is normal, UAG levels are sufficiently high to compensate for the elevated AG levels. However, the UAG levels in patients with PWS from phase 2a onwards are likely to be too low to modulate the effects of elevated AG levels. The resulting higher AG/UAG ratio might induce or contribute to the weight gain and hyperphagia. It is unknown which factors determine UAG levels in healthy subjects and PWS patients. Previous studies showed that somatostatin (agonist) administration did not result in reduction of weight, food intake or appetite [35–37], but it might be that AG and UAG levels are equally affected by this treatment and that the AG/UAG ratio is not influenced. UAG might be secreted via different mechanisms than AG and rates of acylation and/or deacylation of AG might be differently modulated. Also differences in clearance of UAG relative to AG might play a role, but no reports are available. Our findings provide a rationale for a role of relatively decreased UAG levels in the abnormal eating behavior in PWS. It would be interesting to investigate whether a more physiological AG/UAG ratio could be achieved by increasing the plasma UAG or decreasing the plasma AG levels and/or bioactivity, and whether this normalization of the AG/UAG ratio results in a reduction of the hyperphagia.

AG and UAG levels and the AG/UAG ratio at various ages in PWS show a wide variation. In the total group, there was a distinct pattern with higher AG/UAG ratios in the higher nutritional phases than in age-matched controls. For individuals, this implies that a patient with PWS with a higher AG/UAG ratio has a higher chance to be in a higher nutritional phase.

As expected, PWS patients had a more favorable metabolic profile with lower insulin levels and a lower HOMA-IR than obese controls [7], while the IGF-I levels were higher in the PWS than in the obese group. In PWS, we found an inverse correlation between AG and UAG levels and BMI and in addition also an inverse association between UAG levels and HOMA-IR. This suggests that low UAG levels in PWS are associated with less favorable health aspects, such as a higher BMI and insulin resistance. Previous studies have reported similar results in non-PWS subjects, but no inhibitor was added to their blood samples [38–40].

Our study showed no difference in AG/UAG ratios between GH-treated patients and untreated patients with PWS, and there was also no significant association between IGF-I levels and AG/UAG ratio. Thus, in our study, GH treatment seems to have no effect on the AG/UAG ratio, despite earlier notes of Hauffa and Petersenn in which GH-treated was assigned as confounding factor in the natural course of ghrelin concentrations [41]. We assume that this study was not designed to investigate the effects of GH treatment on ghrelin levels.

As blood sampling in children and young adults with PWS is difficult and quite invasive, we collected only one fasting sample. In our opinion, fasting samples are most appropriate and we present the AG and UAG levels of a large group of patients. It would be informative, however, to conduct a longitudinal study in children with PWS, to investigate whether the switch in eating behavior is closely correlated with an increase in the AG/UAG ratio. In addition, it would be of interest to determine whether PWS patients show a postprandial decline in AG and UAG levels.

## Conclusion

We report that PWS patients have higher AG levels but similar UAG levels compared to healthy controls, resulting in a significantly higher AG/UAG ratio in PWS patients than in controls. Obese controls have significantly lower AG and UAG levels than PWS patients and healthy controls, but also a high AG/UAG ratio. The reason for the higher AG/UAG ratio in PWS and obese was, however, completely different, as PWS had a high AG and obese a very low UAG.

PWS patients without weight gain or hyperphagia had a similar AG/UAG ratio as age-matched controls, in contrast to those with weight gain and/or hyperphagia who had an elevated AG/UAG ratio. The switch to excessive weight gain in PWS seems to coincide with an increase in the AG/UAG ratio, even prior to the start of hyperphagia.

## Electronic supplementary material

AG and UAG levels of PWS per nutritional phase and of healthy and obese controls. This boxplot shows the AG (in white) and UAG (in gray) levels of children and young adults with PWS in the 5 nutritional phases and of healthy controls and obese controls. The lower boundary is the 25^th^ percentile and the upper boundary the 75^th^ percentile. The line in the box represents the median. Lines are drawn from the smallest to the largest observed value that is not an outlier (PDF 137 kb)
