# Supplementary figures and images for: Elevated ratio of acylated to unacylated ghrelin in children and young adults with Prader–Willi syndrome
Source: Endocrine. 2015 May 20;50(3):633–42. doi: 10.1007/s12020-015-0614-x (PMC4662713; doi:10.1007/s12020-015-0614-x)

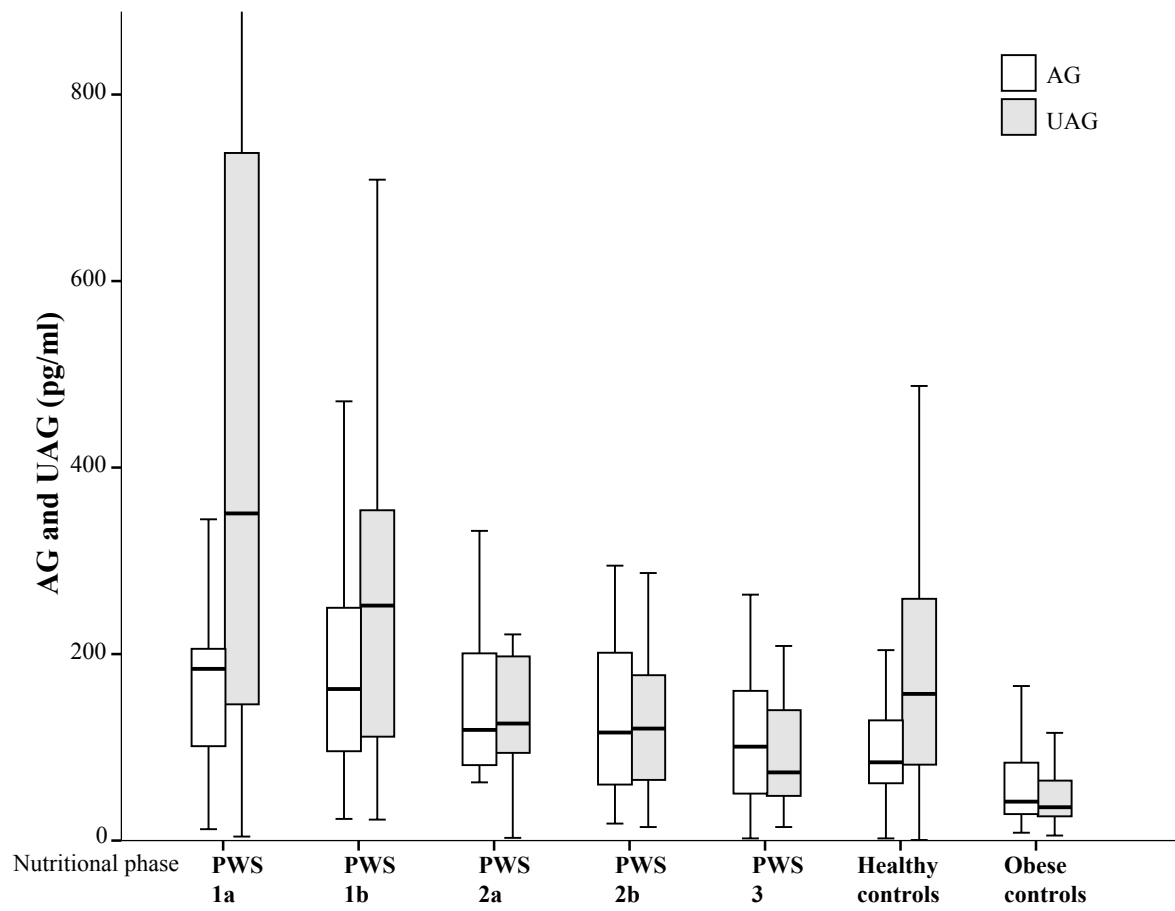

Supplement: Supplementary file 1 — AG and UAG levels of PWS per nutritional phase and of healthy and obese controls. This boxplot shows the AG (in white) and UAG (in gray) levels of children and young adults with PWS in the 5 nutritional phases and of healthy controls and obese controls. The lower boundary is the 25th percentile and the upper boundary the 75th percentile. The line in the box represents the median. Lines are drawn from the smallest to the largest observed value that is not an outlier (PDF 137 kb) [file 12020_2015_614_MOESM1_ESM.pdf]
